# The Effectiveness of Telephone-based Psychological Services to COVID-19

**DOI:** 10.2174/17450179-v19-230824-2023-11

**Published:** 2023-08-30

**Authors:** Mojgan Khademi, Roya Vaziri-Harami, Amin Mahouram Mashadi, Pegah Seif, Abbas Babazadehdezfoly

**Affiliations:** 1 Department of Psychiatry, School of Medicine, Imam Hossein Hospital, Shahid Beheshti University of Medical Sciences, Tehran, Iran; 2 Clinical Research Development Unit, Imam Hossein Hospital, Shahid Beheshti University of Medical Sciences, Tehran, Iran

**Keywords:** Telehealth, Telemental health, Telepsychiatry, COVID-19, PTSD, Psychological disorders

## Abstract

**Background:**

The COVID-19 pandemic has disrupted the delivery of mental health services, leading to the development of telepsychiatry.

**Aim:**

The present study investigates the effectiveness of telephone-delivered treatment for psychological disorders of COVID-19 survivors in Tehran, the capital of Iran.

**Methods:**

In this non-randomized controlled trial, 91 COVID-19 survivors, primarily residents were enrolled. Participants completed a baseline questionnaire and a psychological screening questionnaire. The intervention included the telephone-based psychological services provided by trained psychiatric residents. The Post-Traumatic Stress Disorder (PTSD) Checklist (PCL) was administered to assess the presence of PTSD symptoms. Symptoms of anxiety and depression were measured by the Patient Health Questionnaire.

**Results:**

The General Health Questionnaire (GHQ) adjusted mean difference was significantly lower in the intervention group than in the control group. There was a significant negative correlation between the Spost-GHQ score and history of going to the clinic and history of psychiatric disorders, but no relationship with the history of hospitalization. All participants completed the satisfaction form, with almost half of them being “satisfied” or “very satisfied” with the telehealth calls.

**Conclusion:**

Telephonic delivery of psychological services showed an effective way of providing evidence-based psychological support during the pandemic. This telehealth program can offer much-needed assistance to individuals with COVID-19 improving their psychological wellbeing.

## 1 INTRODUCTION

Coronavirus disease (COVID-19), a novel coronavirus disease, has emerged as a major global health issue. In contrast to past pandemics, this disease is highly contagious and has a faster rate of transmission among individuals [1]. As the number of COVID-19 cases and fatalities continue to rise, an increasing number of patients are reporting psychological distress [2, 3]. Early investigations into the mental health of those who have survived COVID-19 have indicated that they frequently exhibit symptoms of post-traumatic stress disorder (PTSD) [4], depression, and anxiety following hospital discharge [5]. The length of hospitalization, environment, and history of psychiatric disorders are all critical factors that influence the occurrence of mental health problems [6]. Given the psychological impact of COVID-19, it is crucial to address psychiatric complications in those who have survived the disease [7].

Untreated mental symptoms may lead to negative health outcomes and increase the cost of disease management [8]. In Iran, various guidelines and expert consensus have been established to address the psychological impact of COVID-19 [9], including the integration of mental health services such as telehealth, utilizing phone, smartphone apps, or email for disaster psychological interventions [1, 10]. However, the efficacy of telemental health, particularly for PTSD, depression, and anxiety, remains unclear [11]. Additionally, the impact of telemental health on psychological disorders in Iran requires further investigation [12]. Consequently, this study aims to assess the effectiveness of telephone-based psychological treatment for COVID-19 survivors in Tehran, Iran's capital city.

## 2 METHODS

### 2.1 Study Design and Participants

This was a non-randomized trial of telephone-delivered psychological intervention or no intervention performed among COVID-19 survivors who were referred to a referral teaching hospital in Tehran, Iran, between April 20 and June 20, 2020. A short telephone-based questionnaire collected demographic and COVID-19-related psychological dimensions. No personal data, which could allow the identification of participants, was collected, to assurance of privacy. Informed consent was obtained before the start of the study. Participants could take out of the investigation at any step without providing any reason, and no data were kept. Only information from questionnaires that had a complete set of participant responses was measured. This study was approved by the Ethics Committee of the Shahid Beheshti University of Medical Sciences.

### 2.2 Screening

A previously approved psychological screening interview was used to assess the presence or absence of psychological distress, functional impairment, and severe mental preoccupation [10]. The Patient Health Questionnaire-4 (PHQ-4) and Posttraumatic Stress Disorder Checklist for DSM-5 (PCL-5) were used to screen for depression, anxiety, and PTSD.

Each individual who had; a positive answer to at least one screening question, or three or more scores in PHQ-4, or suffered from PTSD according to PCL-5, was included in our intervention.

### 2.3 Intervention

Participants in the intervention group received telephone-delivered therapy twice a week. Those in the control group received training about psychological problems and followed up during the intervention.

A psychiatric interview based on DSM-5 criteria for detecting major depression, anxiety, and PTSD, was done for all the selected cases. If they were diagnosed with these disorders, they were advised to be referred to a psychiatric clinic.

Telephone-based psychological services were provided twice a week by trained psychiatric residents. Typically, 5 to 6 sessions were devoted and lasted 30-45 minutes depending on the applied treatment. Phone call discussions included supportive interventions, simple behavioral activation techniques, controlling and replacing dysfunctional thoughts, control of annoying and repetitive thoughts about COVID-19 and related events, emotion regulation, and problem-solving skills. The control group received only psychiatric training in the first session.

### 2.4 Outcome Measures

#### 2.4.1 Depression and Anxiety Symptoms

Symptoms of depression and anxiety were assessed by the Patient Health Questionnaire for depression and anxiety (the PHQ-4) [15]. Respondents were asked to rate how often they have experienced depressive and anxiety symptoms in the past month using a two-item measure, consisting of core criteria for depression and a two-item measure for anxiety. Individuals with a PHQ-4 score of 3 or above were classified as having symptoms of anxiety and/or depression symptoms [10].

#### 2.4.2 PTSD Symptoms

The Post-Traumatic Stress Disorder Checklist (PCL) questionnaire was used to evaluate the presence of PTSD. The PCL contains 20 items that correspond to the diagnostic criteria for PTSD. Respondents were asked to rate on a 5-point scale (1 = not at all, 5 = extremely) the degree of distress experienced within the past 30 days due to PTSD symptoms. Total scores range from 17 to 85, with 44 being the recommended cutoff for PTSD for community samples [16].

#### 2.4.3 General Mental Health Status

The General Health Questionnaire-28 (GHQ-28) evaluated the psychological situation in four main domains: somatic symptoms, anxiety, social dysfunction, and severe depression.

#### 2.4.4 Participants Satisfaction

The authors created a satisfaction scale to assess participants’ opinions and reactions regarding telephone-based treatment delivery. This scale was used for all participants at the beginning and six weeks after the intervention. The measure consists of 4 items and asks respondents to rate on a 4-point scale (1 = poor, 4 = excellent) their telehealth-based treatment experience. Items included confidentiality of services, helpfulness, and sensitivity of therapist, scheduling of sessions, matching treatment to individual needs, and overall service quality.

#### 2.4.5 Statistical Analyses

The treatment effect (adjusted mean difference) was analyzed using a covariance linear regression model, with the post-treatment score as the response variable, baseline score, age, history of psychiatric disorders, history of hospitalization, and clinical history covariates. Correlations were calculated using Spearman’s rank-sum correlation coefficients. All data were analyzed using Statistical Package for Social Sciences (SPSS) version 26.0. *Statistical significance was set at p < 0.05*.

## 3 RESULTS

### 3.1 Study Population and Baseline Parameters

A total of 602 COVID-19 survivors were investigated, of which 91 people answered positively to at least one screening question or had a depression/anxiety score equal to or above 3. Of these, 57 COVID-19 survivors accepted the psychological intervention and were included in the intervention. In the intervention arm, thirty (53%) of participants were male, and the sample had a mean age of 50.0 (standard deviation [SD] = 10.2) (Table **1**). Baseline psychological parameters (depression, dysfunction, severe mental preoccupation, and psychological suffering) were not significantly different in the investigated groups, except for anxiety which was more prevalent in the intervention arm (Table **2**).

### 3.2 Treatment Outcome

The mean GHQ scores between study arms at baseline and 6 weeks post-intervention are shown in Fig. (**1**).

At the post-intervention follow-up, the adjusted mean difference of GHQ was significantly lower in the intervention arm than in the control arm (19.46 *vs.* 27.00, p = 0.00). There was a significant negative correlation between Post-GHQ score and history of psychiatric disorders (Spearman’s correlation coefficient −0.27, P=0.09), but no relationship with the history of hospitalization.

A mixed analysis of variance test assessed the mental health scores pre and post-intervention over the two groups, and it was concluded that the interaction of the intervention is meaningful over time. Therefore, the intervention successfully alleviated mental health (p<0.001). The mean mental health score among those with prior psychiatric disorders in the pretest was 35.42± 7.72 in the intervention group, and among the control group, it was 31.18± 6.92, and these scores were 19.46± 9.74 and 27± 9.02 respectively for the post-test.

The mean mental health score among those without prior psychiatric disorders in the pretest was 35.82± 10.90 for the intervention group, and among the control group, it was 29.50± 6.02. These scores were 18.16± 9.44 and 25.38± 5.66 respectively for the post-test. Thus for both groups, with and without psychiatric disorders, mental health was meaningfully improved in the intervention group compared to the control group.

### 3.3 Participant Satisfaction

All participants completed the satisfaction form, with almost more than half of completers (57.0%, n = 52) being “satisfied” or “very satisfied” with the telehealth calls.

## 4 DISCUSSION

Iran is among the countries most affected by natural disasters owing to its geographical location and vulnerability to climate change. In recent decades, significant earthquakes, storms, and floods have struck Iran, making it crucial for healthcare authorities to provide medical care during these crises [13]. Telemedicine has been used in various countries to diagnose and treat patients' health conditions following disasters, with several authors supporting its clinical feasibility, cost-effectiveness, and diagnostic accuracy [14]. However, telemedicine has been less frequently employed to provide psychological counseling following disasters such as the COVID-19 pandemic [15].

It is encouraging to hear that a telemental intervention program designed to improve psychological problems in people who survived COVID-19 in Iran has led to statistically significant improvements in psychological symptoms over the follow-up period [16]. It is also worth noting that telepsychiatry is reliable and valid in several psychiatric disorders, and before the COVID-19 pandemic, evidence from studies had already indicated that it could improve mental disorders among adults [17]. The meta-analysis by Bolton *et al*. that examined the role of telemental services in the management of PTSD also supports the usefulness of therapist-assisted internet programs in reducing the severity of anxiety and depression symptoms [18]. These findings highlight the potential of telemental health interventions in improving mental health outcomes, especially in situations where face-to-face interventions may not be feasible or accessible [19].

The results of this study provide evidence for the effectiveness of telemental calls in delivering evidence-based psychological treatment during the pandemic. Reports suggest that telemental interventions have been successful in providing services to COVID-19 patients. China was at the forefront of providing various telemental services during the COVID-19 pandemic to address the mental health needs of clinicians and patients who tested positive for the virus [20].

Early reports from China's telehealth interventions have indicated a high level of interest and acceptance of this mode of healthcare delivery. Similarly, numerous studies from different parts of the world have highlighted the importance of promoting telemental healthcare services and encouraging their use [21]. Alessi *et al*. conducted a study to assess the effectiveness of maintaining a telephone conversation program focused on mental health issues during the COVID-19 pandemic [22]. The results showed that the teleintervention group had a lower prevalence of positive screening for mental health problems compared to the control group. Kahlon *et al*. surveyed to evaluate the effectiveness of a telephone-based intervention for depression and anxiety among adults during the COVID-19 pandemic. The intervention led to a reduction in depression and anxiety and improved the overall mental health of the participant's [23].

Telemental intervention has emerged as a highly suitable and viable option to support healthcare providers, patients, and their families during the pandemic, as evidenced by several studies [24]. Telepsychiatry offers numerous advantages, such as the ability for patients to receive psychiatric assistance from anywhere, comparable effectiveness to in-person care, and reduced travel costs and time [25]. Moreover, telepsychiatry can reduce the risk of infection by limiting person-to-person contact between COVID-19 and healthcare providers [26]. Despite positive feedback from clinicians and patients on telepsychiatry, widespread implementation has been slow and challenging due to concerns about technology limitations, safety, and privacy [27].

Clinicians have expressed concerns regarding payment, regulatory structures, licensure, education and learning issues, and program implementation, which pose challenges to clinical care management and system workflow efficiency [28]. Therefore, addressing these concerns is crucial in overcoming the barriers to the widespread adoption of telepsychiatry [29]. Continuous training of clinicians is necessary to increase their awareness of the risks and benefits of telepsychiatry [30]. Furthermore, promoting continuing medical education programs, integrating telepsychiatry into medical school curriculums, and incorporating residency training in telepsychiatry can help to further encourage its use [31].

The present study has several limitations that should be considered. First, the self-administered scales used to assess mental health problems were administered *via* telephone, which may introduce potential sources of bias. Second, randomization of the group allocation strengthened the impact of our findings. Thirdly, although we observed strong results, it is essential to explore whether extended periods of telephone services could yield additional benefits. Lastly, the generalizability of our findings needs to be thoroughly evaluated across communities with varying degrees of preexisting mental health disorders.

## CONCLUSION

In conclusion, our study suggests that telephone-delivered psychological services have the potential to effectively provide evidence-based psychological support during the pandemic. Further analysis of post-intervention data is necessary to obtain a more comprehensive understanding of the program's effectiveness. Additionally, it is crucial to investigate variables that could influence the efficacy of psychological services and contribute to participant attrition. By addressing these limitations, we can enhance the quality and applicability of telehealth programs for managing psychological disorders among individuals with COVID-19.

## CONTRIBUTORS’ STATEMENT PAGE:

Dr. Roya Vaziri-harami and Dr. Mojgan Khademi: conceptualized and designed the study, drafted the initial manuscript, and reviewed and revised the manuscript.

Dr. Pegah Seif and Dr. Abbas Babazadehdezfoly: Designed the data collection instruments, collected data, carried out the initial analyses, and reviewed and revised the manuscript.

Dr. Amin Mahouram Mashadi: Coordinated and supervised data collection, and critically reviewed the manuscript for important intellectual content.

## Figures and Tables

**Fig. (1):**
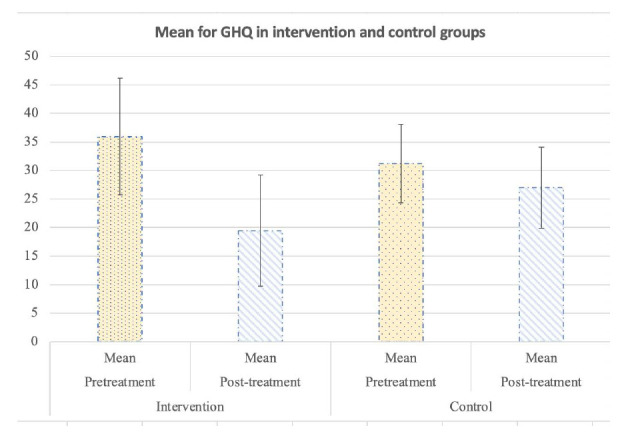
Mean GHQ between intervention and control group.

**Table 1:** Baseline characteristics of participants.

**Baseline Parameters**	**Intervention Group N (%)**	**Control Group,** **N (%)**	***P*-value**
Age (years)	-	-	0.08
<40	11 (19.3%)	7 (20.6%)	-
41-50	20 (35.1%)	6 (17.6%)	-
51-60	18 (31.6%)	10 (29.4%)	-
60-70	5 (8.8%)	3 (8.8%)	-
>70	3 (5.3%)	8 (23.5%)	-
Gender	-	-	0.50
Male	30 (52.6%)	20 (58.8%)	-
Female	27 (47.4%)	14 (41.2%)	-
Total number	57	34	-

**Table 2:** Previous psychological statements of participants.

**Parameters**	**Intervention Group N (%)**	**Control Group, N (%)**	***P*-value**
History of psychiatric disorders	-	-	0.70
Yes	12 (21.1%)	8 (23.5%)	-
No	45 (78.9%)	26 (76.5%)	-
History of hospitalization	-	-	0.50
Yes	42 (73.7%)	27 (79.4%)	-
No	15 (26.3%)	7 (20.6%)	-
History of going to the clinic	-	-	-
Yes	19 (33.3%)	-	-
No	38 (66.7%)	-	-
Anxiety	-	-	-
Yes	23 (40.4%)	3 (8.8%)	0.00
No	34 (59.6%)	31 (91.2%)	-
Depression	-	-	0.60
Yes	18 (31.6%)	9 (26.5%)	-
No	39 (68.4%)	25 (73.5%)	-
Dysfunction	-	-	0.70
Yes	13 (22.8%)	9 (26.5%)	-
No	44 (77.2%)	25 (73.5%)	-
Severe mental preoccupation	-	-	-
Yes	25 (43.9%)	9 (26.5%)	0.10
No	32 (56.1%)	25 (73.5%)	-
Psychological suffering	-	-	0.16
Yes	25 (43.9%)	20 (58.8%)	-
No	32 (56.1%)	14 (41.2%)	-

## Data Availability

Data sharing is not applicable to this article as no datasets were generated or analyzed during the current study.

## LIST OF ABBREVIATIONS

PTSD: = Post-traumatic stress disorder
GHQ: = General health questionnaire
